# A Model of the Ectomycorrhizal Contribution to Forest Soil C and N Dynamics and Tree N Supply Within the EFIMOD3 Model System

**DOI:** 10.3390/plants14030417

**Published:** 2025-01-31

**Authors:** Oleg Chertov, Pavel Frolov, Vladimir Shanin, Irina Priputina, Sergey Bykhovets, Anna Geraskina

**Affiliations:** 1 Center for Forest Ecology and Productivity of the Russian Academy of Sciences, Profsoyuznaya st., 84/32, bld. 14, 117997 Moscow, Russia; shaninvn@gmail.com (V.S.); angersgma@gmail.com (A.G.); 2 Institute of Physicochemical and Biological Problems in Soil Science, Pushchino Scientific Center for Biological Research of the Russian Academy of Sciences, Institutskaya st., 2, 142290 Pushchino, Russia; frolov@pbcras.ru (P.F.); priputina@pbcras.ru (I.P.); s_bykhovets@rambler.ru (S.B.)

**Keywords:** ectomycorrhiza, modeling, growth, N mining, dissolved organic matter, mycelial litter, decomposition, food web fauna, ${NH}_{4}^{+}$, faunal casts

## Abstract

Mycorrhizal symbiosis has been the focus of research for more than a century due to the positive effect of fungi on the growth of the majority of woody plants. The extramatrical mycelium (EMM) of ectomycorrhiza (EMR) accounts for up to one-third of the total soil microbial biomass, whereas litter from this short-living pool accounts for 60% of the total litterfall mass in forest ecosystems. The functioning of EMR improves the nitrogen (N) nutrition of trees and thus contributes to the carbon (C) balance of forest soils. The model presented here is an attempt to describe these EMR functions quantitatively. It calculates the growth of EMM and the subsequent “mining” of additional nitrogen from recalcitrant soil organic matter (SOM) for EMR growth, with the associated formation of “dissolved soil carbon”. The decomposition of EMM litter is carried out by all organisms in the soil food webs, forming available ${NH}_{4}^{+}$ in the first phase and then solid-phase by-products (excretes) as a new labile SOM pool. These substances are the feedback that determines the positive role of EMR symbiosis for forest vegetation. A sensitivity analysis revealed a leading role of the C:N ratio of biotic components in the dynamics of EMM. The model validation showed a satisfactory agreement between simulated and observed data in relation to EMM respiration in larch forest plantations of different ages. Model testing within the EFIMOD3 model system allowed a quantitative assessment of the contribution of different components to forest soil and ecosystem respiration. The validation and testing of this model demonstrated the adequacy of the theoretical background used in this model, with a fast EMM decomposition cycle by all soil biota of the food webs and without direct resource exchange between plants and fungi.

## 1. Introduction

The development of mathematical modeling in ecology, soil science, forestry, and other biological disciplines began in the 20th century with very simple functions, followed by a steady increase in the complexity of the model structure as new experimental data and ideas for the quantification and parameterization of processes in natural systems emerged. The first equations for the dynamics of organic debris and soil organic matter (SOM) described the formation/accumulation of SOM, but then the focus shifted to the processes of SOM decomposition. At that time, the development of SOM models had been focused on SOM formation as an important factor for plant nutrition and the improvement of edaphic conditions in terrestrial ecosystems [1,2,3]. Nowadays, the focus has shifted back to the sequestration of “soil organic carbon” (SOC instead of SOM) in the soil system as part of global carbon-cycle research [4,5,6,7].

The number of recent models of SOM dynamics is quite large (see the review in [8]). The effect of biological factors in recent models is mostly limited by the microbiological activity. Some recent SOM models are integrated with plant/vegetation simulators and reproduce the organic carbon cycle at the ecosystem level [9,10,11]. This has led to an increased interest in the reasonable studying of the intrinsic biotic mechanisms of “plant–soil biota–soil” interactions.

Two widespread mechanisms of “plant–soil” biotic interactions are (i) the activation of rhizosphere soil microorganisms via plant roots, the so-called “priming effect” [12,13], and (ii) the symbiosis of plant roots with mycorrhizal fungi [14,15,16]. Symbiotic microbial nitrogen fixation and nutrient transfer from the canopy to the soil through litterfall are also such “plant–soil” interactions [17,18,19]. The cumulative effect of these mechanisms of “plant–soil biota–soil” interactions leads to the optimization of the edaphic environment in terrestrial ecosystems and the increased growth and vitality of plants.

The rhizosphere priming effect is well documented and has been studied in detail [12], which has allowed the mathematical simulation of this phenomenon [20,21]. However, mycorrhizal symbiosis is a more powerful type of “plant–soil biota–SOM” interaction that has been underexplored for about 150 years. It is a widespread phenomenon of the world’s vegetation. There are five types of such symbioses [14]: ectomycorrhiza, arbuscular, ericoid, orchid, and mycoheterotrophic mycorrhizae. However, only two types dominate globally: ectomycorrhiza in temperate and boreal forests and arbuscular mycorrhiza in forests and grasslands, mostly in warmer climates.

Ectomycorrhiza (EMR) forms a mycelial mantle (sheath) on the growing root tip that produces a large amount of extramatrical mycelium (EMM), which penetrates the entire volume of organic and organomineral soil horizons. Arbuscular mycorrhizal cells live inside the roots from where the EMM hyphae occupy a large soil volume. EMR explores the entire soil volume, and its dense network of EMM “mines” the nutrients from the SOM [22].

Mycorrhizal fungi are obligate biotrophs that feed on matter and energy received from the host plant in the form of root exudates (REs). The products of plant photosynthesis are the driving force behind the functioning of EMR. Trees spend 10–25% of their net primary production (NPP) on REs [23]. REs include carbohydrates, amino acids, organic acids, and some other organic compounds [14,15,24]. However, due to an imbalance of C and N in their composition, REs do not cover all EMM growth requirements [25]. EMR performs a rather specific pattern of SOM decomposition, replenishing N via “mining” from the SOM [14,23,26,27]. EMM depolymerizes large organic molecules of N-rich recalcitrant SOM for N extraction (“mining”), but it does not mineralize all the “dissolved organic carbon” (DOC) obtained. This is a major difference between mycorrhiza and soil saprotrophic fungi: EMM produces a lot of DOC [22,28], but it contributes about 2% to the total SOM mineralization [29,30,31]. This DOC after N mining, plus the rest of the acidic REs after the mycorrhizal consumption of carbohydrates and amino acids, is a biochemical agent in the dissolution of soil minerals [32,33].

EMM has a short lifespan, varying from a few days to several weeks [14,34,35]. This results in a high turnover rate of EMM. Therefore, EMM produces a large litter pool, which is larger than leaf litter and can be up to 62% of the total annual above- and belowground litter of trees [36,37,38,39]. The decomposition of this large and previously unknown pool of fungal litter is carried out by all soil biota: bacteria, fungi, and the micro- and mesofauna of food webs. However, the role of soil fauna in the future of this specific litter is not addressed in most EMR studies [40], resulting in an underestimation of the contribution of EMM litter to SOM formation. In the case of pure microbial decomposition, the intermediate product of the microbial exoenzymatic depolymerization of SOM is DOC. It is consumed by microorganisms for their own biomass growth [41]. Nevertheless, there is a concept in soil science that DOC produced through microbial activity is an important source of SOM formation through stabilization on the surface of soil minerals [42,43,44].

The situation is different in the case of mycelial litter decomposition with the participation of the fauna of soil food webs as an active agent of litter transformation [45]. Soil micro- and mesofauna consume litter, transform organic matter, and produce solid-phase metabolic products of casts, which have been known in soil ecology since the 19th century as the main products of SOM formation [46,47]. These casts represent a previously unrecognized labile SOM fraction. Some excess N is produced during the consumption of microorganisms (including EMM) by soil fauna, as a significant part of the C consumed is used for faunal respiration [48,49]. This pathway of EMM litter transformation and decomposition is consistent with experimental data on the prominent role of food webs in resource acquisition and consumption in soils of terrestrial ecosystems [21,48,49,50,51,52,53]. With regard to soil food webs, their active role has been confirmed by oscillatory respiration on an hourly scale, which reflects the high activity of the “predator–prey” type of relationships between the soil fauna of “microbial grazers” and microorganisms [54,55]. This role of EMR in SOM recovery and sequestration represents feedback in the mycorrhizal symbiosis that determines a well-known positive effect of EMR on tree growth [56,57]. Thus, the increase in the biomass increment, the photosynthetic rate, or respiration in tree seedlings with ectomycorrhiza was 1.2–2.6 times higher than that of non-infected plants [14,58,59,60].

Currently, there is a growing interest in the mathematical modeling of the functioning of EMR. However, EMR is a rather complicated object for simulation modeling because it is structurally and functionally linked to both the growth of trees and SOM dynamics. In forest ecosystems, EMR lies in between autotrophic and heterotrophic systems, being part of both. There are studies on the principles of and approaches to EMR simulation [61,62,63], the modeling of special patterns of EMM distribution in forest ecosystems [64], the model assessment of nitrogen uptake by EMR fungi [4], the quantification of factors of EMM decomposition [65], and the well-parameterized simulation of EMM growth [66]. More promising is a successful attempt to integrate the EMR model into MYCOFON, the whole-ecosystem simulator [67].

The majority of recent EMR models have two specific features: (i) they consider the dynamics of EMM litter decomposition as influenced only by microorganisms, without taking into account the soil fauna of food webs; (ii) the models are not integrated into whole-ecosystem models. We carried out the “mining” of scientific publications for experimental data with their synthesis and conceptualization, which allowed us to draw conclusions about the significant role of EMR decomposition for C and N dynamics and the tree N supply [40]. Therefore, the aim of this study was to develop an EMR model that can reproduce EMR growth and decomposition with the role of all soil biota and with special reference to soil food webs, SOM dynamics, and plant nutrition, namely to determine the following: (i) the role of soil fauna in extramatrical mycelium decomposition; (ii) the role of EMR in nitrogen release for plant growth; (iii) the role of EMR in soil respiration; and (iv) the contribution of EMR to the SOM formation. This model was developed for integration with the Romul_Hum model of SOM dynamics [8,53] as part of the latest version of the model system, EFIMOD3 [68].

## 2. Materials and Methods

### 2.1. Model Description

#### 2.1.1. Basic Postulates

The following postulates, based on previous studies on the functioning of the EMM, were used to construct the model. In mycorrhizal symbiosis, the fungi are very active and consume all the N of the REs for their own growth (mainly for the EMM), making the transfer of N of exudates to the soil through the dense Hartig net of the mycelial mantle impossible.

EMM decomposes SOM to cover the N deficiency for EMM growth only. This is selective N mining from recalcitrant stable SOM with increased N concentration. In effect, the mycorrhizal fungi are de facto “working for themselves”.

EMM has a short lifespan. Therefore, it produces a large litter flux, which is supposed to be a dominant component in the structure of total litter input in forest ecosystems. This large litter flux of EMM is consumed by soil micro- and mesofauna of different trophic levels in soil food webs. It ensures SOM replenishment via solid-phase products of soil faunal metabolism, namely labile SOM from faunal excreta. In addition, the consumption of EMM litter by microfauna (“microbial grazers”) results in the formation of ammonia N.

Additional N in EMR is obtained through N mining during selective decomposition of recalcitrant SOM and also after EMM consumption by soil fauna in food webs. This N becomes available to roots after microbial decomposition of rapidly decomposing N-rich labile SOM that originated from faunal excreta. This explains the positive role of EMR in tree nutrition and growth.

#### 2.1.2. Initialization

The spatially explicit process-based EFIMOD3 system of models [68] is a further development of the well-known EFIMOD2 model [10]. It focuses on the simulation of spatial heterogeneity in forest ecosystems due to the vegetation structure and “plant–environment” interactions. EFIMOD3 has a discrete time step of 1 day. The simulation plot with a maximum possible size of 1 ha (100 × 100 m) is divided into cells with a default size of 0.5 × 0.5 m. The system of models is implemented in R v. 4.4.2 [69]. It consists of several models.

(i) *The initial generation of the microrelief* is based on the construction of a 2-dimensional array of cells with defined statistical properties of the distribution of their relative altitudes. The model allows the setting of spatial heterogeneity at different scales and general slopes. (ii) *The initial location of trees* is based on a set of different algorithms and can be of several types: pseudo-random uniform, pseudo-random with hardcore distance, regular, clustered, or gradient. (iii) *The inter-tree competition for the photosynthetically active radiation* (PAR) model generates the asymmetric shape of tree crowns and the distribution of biomass within them, depending on the exact position and size of the competing trees. (iv) *The inter-tree competition for soil nitrogen* model simulates the distribution of the belowground biomass of trees, depending on the amount of available nitrogen in the soil, the biomass of the roots of competing trees, and the distance from the stem base. The model also describes the vertical distribution of belowground biomass. These models are individual-based and spatially explicit, and they are able to simulate the plasticity of crown and root systems resulting from the heterogeneity of biotic and abiotic environments. (v) *A process-based model of tree biomass production* reproduces the net primary production, growth, and maintenance respiration and evapotranspiration of individual trees. The model has species-specific coefficients and calculates outputs as a function of PAR and the available nitrogen, stochastic tree mortality, and the spatial distribution of litterfall. (vi) *The allocation of tree biomass between compartments* (stem, branches, foliage, and structural and fine roots) is described by a rank distribution function with species-specific and age-dependent coefficients. (vii) *The ground layer vegetation* model calculates the species-specific biomass increment, mortality, litterfall production, regeneration, and dispersal of plants. (viii) *Soil hydrothermal conditions* are simulated by calculating heat and water transfer through the soil layers, as well as lateral water flows in the soil. (ix) *Soil organic matter dynamics* is described by a Romul_Hum model [8,53], which calculates the decomposition and humification rates of fresh litter, forest floor, and labile and stable SOM as a function of soil hydrothermal conditions and the SOM nitrogen and ash content. The model simulates the activity of soil micro- and mesofauna of food webs, heterotrophic soil respiration, and the nitrogen available to plants, thus reproducing the feedback from soil to vegetation.

The EMR model has been developed for steady-state conditions with developed mycorrhiza in a forest ecosystem, where mycorrhizal growth is in equilibrium with its die-off and consumption by soil fauna (faunal consumption is a dominant process), and it is linked to the growth of fine roots. All calculations in the model are performed in terms of carbon stocks per unit area (e.g., in kg m^−2^), with a time step of 1 day. Hydrothermal conditions are assumed to be optimal with soil moisture at 60% of the specific water retention and a soil temperature of 20 °C. Total SOC is the sum of the carbon stocks of the organic and organomineral horizons (O and Ah/AhE) in the topsoil. The pools of SOM-C, SOM-N, the biomass of fine roots, and the amount and composition of REs are obtained from the EFIMOD3 system of the models within which the EMR model was developed. All parameters and variables are presented in Table 1 and Table 2.

The biomass of the ectomycorrhizal mantle accounts for 30–60% of the fine-root biomass [14,73,74]. We postulated it as a constant fraction of 0.4 of the fine-root biomass of trees. To date, the fraction of fine roots determined experimentally in the field studies includes both roots and the ectomycorrhizal mantle. It follows that a fraction of the ectomycorrhizal mantle biomass can be isolated from the experimentally measured fine-root biomass. Therefore, we used the fine-root biomass to determine the initial value and dynamics of the fungal mantle in the EMR model, which had been integrated into the EFIMOD3 system of models. It is included in the model formulation as part of the ectomycorrhizal biomass. The lifespan of the mantle is 2–3 weeks, while the lifespan of fine roots without a mantle is about 3 years [14].

Ectomycorrhiza develops a dense network of EMM, which also has the same lifespan as the mantle [14,34,35,75]. Its biomass accounts for 30% of the total biomass of all soil microorganisms [22], and this parameter was used to calculate the initial biomass of EMM. In the absence of measured data, the biomass of soil microorganisms can be estimated using the equation derived from [76]:

(1) $$C_{mic}=3.91-0.30\times C_{SOM},$$

where $C_{mic}$ is the C of all microorganisms (all fungi and all bacteria, but excluding microfauna), the % of total SOC ($C_{SOM}$).

The biomass of EMM ($C_{EMM}$) can be calculated as

(2) $$C_{EMM}=0.3\times C_{mic}.$$

The C:N ratio of the EMM ($CN_{EMM}$) is also required to calculate the EMM dynamics in the model. It can be calculated using the following equation [53]:

(3) $$CN_{EMM}=0.85\times CN_{SOM},$$

where $CN_{SOM}$ is the C:N ratio of soil organic matter.

#### 2.1.3. Root Exudates Input

The input of root exudates ($C_{RE}$, kg [C] m^−2^ day^−1^) is determined in the productivity model of EFIMOD3 as a part of the gross primary production (GPP) of trees; alternatively, this input can be obtained from the available measured data. The actual REs consumption (${∆C}_{RE}^{cons}$) can be partially reduced due to competition with priming ($k_{pr}^{EMM}$) and the REs consumption efficiency ($k_{RE}^{cons}$):

(4) $${∆C}_{RE}^{cons}=C_{RE}\times k_{pr}^{EMM}\times k_{RE}^{cons}.$$

The second and third terms of this equation are assumed to be 1.0 in this pilot version of the model due to the lack of experimental data.

#### 2.1.4. EMM Growth

The consumed REs are used for EMM growth (${∆C}_{EMM}^{RE}$) taking into account (i) the N pool in REs ($N_{RE}$) and (ii) the need for REs carbon for EMM growth respiration. It can be expressed by two equations:

(5) $${∆C}_{EMM}^{RE}=N_{RE}\times {CN}_{EMM},$$

where ${CN}_{EMM}$ is the C:N ratio of the EMM biomass; and

(6) $$R_{EMM}={∆C}_{EMM}^{RE}\times \frac{k_{EMM}^{R}}{k_{EMM}^{Gr}},$$

where $R_{EMM}$ is EMM respiration as a cost of EMM growth, $k_{EMM}^{Gr}$ is a proportion of the consumed C spent on EMM growth, and $k_{EMM}^{R}$ is a proportion of the consumed C spent on respiration (with the condition that $k_{EMM}^{Gr}+k_{EMM}^{R}=1.0$).

The use of REs for EMM growth and respiration does not utilize the entire REs pool due to insufficient nitrogen content (the C:N ratio of REs is significantly higher than the C:N ratio of EMM biomass). The rest of the REs (${rest}_{RE}$) is calculated as follows:

(7) $${rest}_{RE}={∆C}_{RE}^{cons}-R_{EMM}-{∆C}_{EMM}^{RE}.$$

This pool contains a significant amount of organic acids, as REs proteins and carbohydrates are consumed by EMM for biomass production and respiration.

#### 2.1.5. N Mining

The complete assimilation of REs carbon for EMM growth requires additional nitrogen, which is “mined” by fungi from the SOM by decomposing it down into soluble compounds (dissolved organic carbon, DOC) during the process of the enzymatic depolymerization of large organic molecules [15,27]. It is necessary to determine the N deficiency for the assimilation of the rest of the REs for EMM growth (${∆N}_{mng}$):

(8) $${∆N}_{mng}=\frac{{rest}_{RE}}{{CN}_{EMM}}.$$

It is possible to calculate the amount of SOC that is “mined” from the total pool of SOC (∆DOC) using the following equation:

(9) $$∆DOC={∆N}_{mng}\times {CN}_{SOM},$$

where ${CN}_{SOM}$ is the C:N ratio of the total SOM. This SOC represents depolymerized soluble organic matter with a greatly reduced N content after its “mining” for the assimilation of the remaining C of REs for EMM growth:

(10) $${∆C}_{EMM}^{mng}={∆N}_{mng}\times {CN}_{EMM},$$

where ${∆C}_{EMM}^{mng}$ is the additional growth of EMM using “mined” N and the rest of the REs.

The processes of REs assimilation for EMM growth and N mining from total SOM lead to the production of two fractions of nitrogen-poor and acidified soluble organic matter. This mixture represents dissolved organic carbon (DOC) at a given time step, *i*, which can be described by the following equation:

(11) $$DOC^{i}={DOC}^{i-1}+{rest}_{RE}-{∆C}_{EMM}^{mng}+∆DOC.$$

#### 2.1.6. EMM Consumption and Mortality

The biomass of EMM decreases under the influence of natural mortality and consumption by the soil fauna of food webs:

(12) $${∆C}_{EMM}^{mort}=C_{EMM}\times k_{EMM}^{mort},$$

(13) $${∆C}_{EMM}^{cons}=C_{EMM}\times k_{EMM}^{cons},$$

where ${∆C}_{EMM}^{mort}$ is the EMM mortality, ${∆C}_{EMM}^{cons}$ is the consumption of EMM by soil micro- and mesofauna in food webs, and $k_{EMM}^{mort}$ and $k_{EMM}^{cons}$ are the rates of natural mortality and consumption by soil fauna, respectively.

The total carbon budget of EMM at a given time step *i* ($C_{EMM}^{i}$) can be expressed as the difference between the corresponding value at a previous time step ($C_{EMM}^{i-1}$) and all incoming and outgoing fluxes, taken with the corresponding sign:

(14) $$C_{EMM}^{i}=C_{EMM}^{i-1}+{∆C}_{RE}^{cons}-R_{EMM}-{∆C}_{EMM}^{mort}-{∆C}_{EMM}^{cons}.$$

According to the model formulation, the EMM necromass becomes a resource for microbiological decomposition, as postulated in microbiological models of SOC dynamics [5,20].

#### 2.1.7. Food Webs’ Dynamics

The main consumers of EMM biomass are the soil micro- and mesofauna of food webs. A specific feature of soil fauna physiology is the formation of solid excreta as a major metabolic by-product. This process is important for understanding the role of micro- and mesofauna in the soil carbon balance. Cast excretion (${∆C}_{MEF}^{excr}$), respiration ($R_{MEF}$), and the growth (${∆C}_{MEF}$) of micro- and mesofauna can be described by an operation of the value of EMM biomass consumed, the cast production rate ($k_{MEF}^{excr}$), and the respiration rate ($k_{MEF}^{R}$):

(15) $${∆C}_{MEF}^{excr}={∆C}_{EMM}^{cons}\times k_{MEF}^{excr},$$

(16) $$R_{MEF}={∆C}_{EMM}^{cons}\times k_{MEF}^{R},$$

(17) $${∆C}_{MEF}={∆C}_{EMM}^{cons}-{∆C}_{MEF}^{excr}-R_{MEF}.$$

These three equations describe the functioning of the soil fauna in relation to the use of EMM as a basic level of food webs, although, in other cases, the basic level is represented by saprotrophic microorganisms.

In this case, part of the EMM consumed is used for respiration, while some N remains in excess and is excreted as ammonium (${∆N}_{MEF}^{min}$) [48,49]. This can be calculated using the following equation ([21,48], adopted):

(18) $${∆N}_{MEF}^{min}=\frac{{∆C}_{EMM}^{cons}}{CN_{MEF}}-\frac{{∆C}_{EMM}^{cons}}{CN_{EMM}},$$

where $CN_{MEF}$ is the C:N ratio of micro- and mesofauna. The proportion of the faunal population ($C_{MEF}$) that die off is described as follows:

(19) $${∆C}_{MEF}^{mort}=C_{MEF}\times k_{MEF}^{mort},$$

where ${∆C}_{MEF}^{mort}$ is the faunal necromass production (in terms of carbon), and $k_{MEF}^{mort}$ is the faunal mortality rate.

Micro- and mesofauna are a food resource for higher trophic levels with larger predators in soil food webs. In this case, the consumption of micro- and mesofauna can be represented as follows:

(20) $${∆C}_{MEF}^{cons}=C_{MEF}\times k_{MEF}^{cons},$$

where ${∆C}_{MEF}^{cons}$ is the consumed biomass (in terms of carbon) of micro- and mesofauna, and $k_{MEF}^{cons}$ is the consumption rate.

An overall carbon budget of the trophic level of micro- and mesofauna in food webs at a given time step *i* can be represented as follows:

(21) $$C_{MEF}^{i}=C_{MEF}^{i-1}+{∆C}_{EMM}^{cons}-R_{MEF}-{∆C}_{MEF}^{excr}-{∆C}_{MEF}^{mort}-{∆C}_{MEF}^{cons}.$$

The representation of the higher trophic levels of food webs (soil macrofauna) in the current version of the model does not include aboveground fauna and earthworms. The macrofauna consumes the biomass of the micro- and mesofauna. The fluxes associated with the soil macrofauna are described in the same terms as for the micro- and mesofauna:

(22) $${∆C}_{MAF}^{excr}={∆C}_{MEF}^{cons}\times k_{MAF}^{excr},$$

(23) $$R_{MAF}={∆C}_{MEF}^{cons}\times k_{MAF}^{R},$$

(24) $${∆C}_{MAF}={∆C}_{MEF}^{cons}-{∆C}_{MAF}^{excr}-R_{MAF},$$

(25) $${∆N}_{MAF}^{min}=\frac{{∆C}_{MEF}^{cons}}{CN_{MAF}}-\frac{{∆C}_{MEF}^{cons}}{CN_{MEF}},$$

(26) $${∆C}_{MAF}^{mort}=C_{MAF}\times k_{MAF}^{mort},$$

(27) $${∆C}_{MAF}^{cons}=C_{MAF}\times k_{MAF}^{cons},$$

(28) $$C_{MAF}^{i}=C_{MAF}^{i-1}+{∆C}_{MEF}^{cons}-R_{MAF}-{∆C}_{MAF}^{excr}-{∆C}_{MAF}^{mort}-{∆C}_{MAF}^{cons}.$$

**Figure 1:**
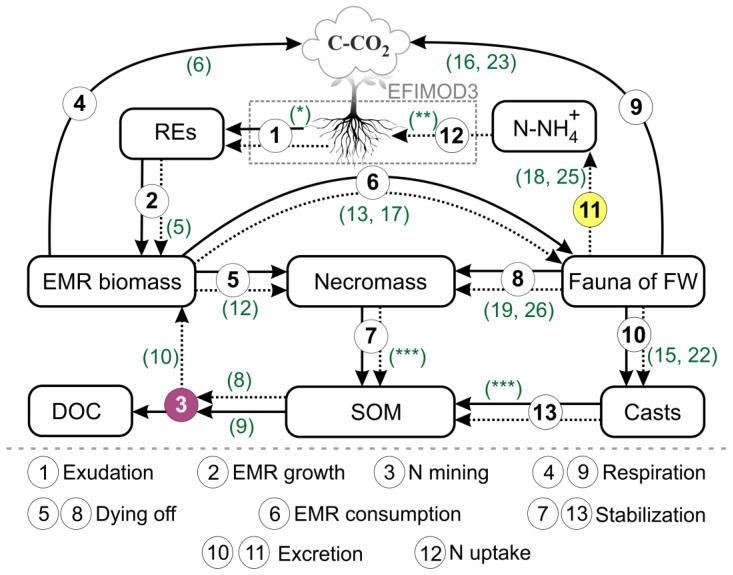
Flowchart of the model of EMR functioning in forest soils. The solid lines represent C fluxes, and the dotted lines represent N fluxes. The dashed rectangle represents the integration with the EFIMOD3 system of models. The numbers in the circles represent the processes of EMR functioning reproduced in the model. The numbers in parentheses near the arrows refers to the equations (see the text) describing the corresponding processes. The purple and yellow circles indicate features that are exclusively represented in the proposed model. * The amount of REs calculated in EFIMOD3 (external input; currently assumed as a proportion of GPP). ** Feedback to EFIMOD3 calculated as a function of tree fine root biomass and species-specific uptake rate. *** These fast-decomposable fractions are assumed to be the intrinsic components of SOM, with their further decomposition to be described in the Romul_Hum model as a part of EFIMOD3. The following abbreviations are used in the flowchart: REs are the root exudates; EMR is the ectomycorrhiza; FWs are the food webs; DOC is the dissolved organic carbon; SOM is the soil organic matter; N is the nitrogen; C-CO_2_ is the carbon dioxide emission in terms of carbon; and N-$NH_{4}^{+}$ is the ammonium excretion in terms of nitrogen.

The outputs of the model are listed in Table 1. This list can be modified according to the specifics of the simulation tasks. The general scheme of the model is shown in Figure 1.

### 2.2. Sensitivity Analysis

The analysis of model sensitivity to uncertainty in parameter estimation was carried out in the same way as for the previously published priming model [21]. The approach of testing sensitivity to each parameter separately is often criticized for not being able to account for the complexity arising from possible non-linear interactions. Therefore, we used the approach proposed in [77]. According to this approach, we set the variation of each input parameter to the ±30% deviation from the default value (shown in Table 2), thus obtaining three values for each parameter. A dataset containing all possible combinations of parameter values was then compiled. The model was run for one year (365 daily time steps) for each record in the dataset, and the sum of EMM, soil micro-, meso-, and macrofauna respiration (${R_{EMM}+R}_{MEF}+R_{MAF}$) was calculated as the target variable, whose calculation involves (either directly or indirectly) all analyzed parameters. Then, the multiple linear regression analysis of the standardized values ($x_{i}^{S}$)

(29) $$x_{i}^{S}=\left(\frac{x_{i}-\bar{x}}{S(x)}\right)$$

of the output variable with respect to the set of standardized values of the input parameters (*I*_1_ … *I_n_*) was performed, where $x_{i}$ is the input parameter or target variable, $\bar{x}$ is the mean, and S(x) is the standard deviation. In this analysis, the higher the absolute value of the regression coefficient *c*_0_ … *c_n_* (the subscript 0 refers to an intercept) associated with a given parameter (as listed in Table 3), the greater the contribution of that parameter to the model output, while the value of the coefficient of determination (R^2^) indicates the non-linearity of the model (the lower the R^2^, the higher the non-linearity).

### 2.3. Model Validation

Despite a very large number of publications on mycorrhizal symbiosis (cited in [14,15]), papers on EMR dynamics with a complete dataset of environmental and physiological parameters of EMR functioning in forest ecosystems are not as abundant. We found four papers with sufficiently detailed data on mycorrhizal respiration, soil climate, EMM biomass production, and soil properties. All studies were carried out on the same experimental plot at the same time. This allowed us to reconstruct all the input parameters and data necessary for the validation of the EMR model.

The comprehensive EMM respiration data from [78] were used to validate the model. In this paper, the average EMM respiration values for the spring, summer, and autumn months are given for *Larix gmelinii* var. *principis-rupprechtii* plantations of 3 different ages (11, 25, and 45 years) located in northeastern China. Data from [79] were used to reconstruct the seasonal dynamics of mycorrhizal respiration. In this paper, the seasonal dynamics of heterotrophic and autotrophic respiration (including mycorrhizal respiration), soil temperature, and soil moisture are provided with a time step of 2 weeks. Data from [79] were interpolated to daily resolution using a cubic spline method [80]. The seasonal dynamics of mycorrhizal respiration were calculated in proportion to the seasonal dynamics of autotrophic respiration:

(30) $$R_{EMM}^{DOY}=\frac{R_{A}^{DOY}}{\sum R_{A}^{DOY}}\times \sum DOY^{0}\times \bar{R_{EMM}^{DOY}},$$

where DOY is the Julian date ($\sum DOY^{0}$ denotes the total number of days for which the measured data are available), $R_{EMM}^{DOY}$ is the measured ectomycorrhizal respiration at *DOY*, $R_{A}^{DOY}$ is the measured autotrophic respiration at DOY, and $\bar{R_{EMM}^{DOY}}$ is the mean daily ectomycorrhizal respiration.

Net primary production (NPP) data from different tree organs for these plantations from [81] were used to create an input dataset on the amount of root exudates entering the soil. The global root exudates flux is estimated to be about 9% (7–14%) of the global annual gross primary productivity (GPP) for mature trees [82]. At the same time, a number of studies estimate NPP to be 0.47 ± 0.04 of GPP [83]. As stated in [84], the production of root exudates is proportional to the proportion of total NPP used for root growth. Therefore, the following equation was used to calculate root exudates (*RE*) production for 3 scenarios of exudates production rate and 3 stand ages:

(31) $$RE=\frac{NPP_{tot}^{age}}{0.47}\times RE_{prt}\times \frac{NPP_{roots}^{age}\times NPP_{tot}^{mature}}{NPP_{roots}^{mature}\times NPP_{tot}^{age}},$$

where $NPP_{tot}^{age}$ is the total NPP for stands aged 11, 25, or 45 years, $RE_{prt}$ is the proportion of root exudates in total GPP for 3 different scenarios of exudates production (0.07, 0.09, and 0.14, respectively), $NPP_{roots}^{age}$ is the NPP of roots for stands aged 11, 25, and 45 years; $NPP_{tot}^{mature}$ is the total NPP for a stand aged 45 years, and $NPP_{roots}^{mature}$ is the NPP of roots for a stand aged 45 years.

To reconstruct the seasonal dynamics of the root exudates supply, the daily NPP was assumed to be proportional to the amount of mineralized soil N. The dependence of the mineralization rate on soil temperature and moisture from the Romul_Hum model [8] was used to calculate the seasonal dynamics of N mineralization and hence the root exudates supply. Finally, the ectomycorrhizal respiration calculated via the model was compared with measured data from [78] using Theil’s U index of inequality (0…1; [85]):

(32) $$U=\frac{\sqrt{\frac{1}{n}\times \sum_{1}^{n}{\left(o_{t}-m_{t}\right)}^{2}}}{\sqrt{\frac{1}{n}\times \sum_{1}^{n}o_{t}^{2}}+\sqrt{\frac{1}{n}\times \sum_{1}^{n}m_{t}^{2}}}\;,$$

where *n* is the number of observations, *o_t_* is the observed EMM respiration at day *t*, and *m_t_* is the simulated EMM respiration at day *t.* The case where U = 0 corresponds to fully agreement between measured and simulated values.

### 2.4. Testing the EMR Model

The EMR model was tested using data from the 1 ha (100 × 100 m) permanent experimental sample plot in a mixed uneven-aged forest (*Pinetum myrtillo-oxalidosum*) dominated by *Pinus sylvestris* L., *Betula* spp., and *Picea abies* L. on Raw Humus Albic Luvisol loamy sand of the well-drained Oka River terrace. The forest community is located in the Prioksko-Terrasny Biosphere Reserve, south of the Moscow Region, Russia (54.88876° N, 37.56273° E). All trees with a stem diameter at breast height (DBH) greater than 6 cm were mapped, and the species, height, DBH, and crown dimensions were recorded. Soil was sampled in 35 small trenches, and soil C and N stocks were estimated separately for the forest floor and the top 30 cm of mineral soil. These data were used for model initialization. A more detailed description of the sample plot can be found in [68,86].

The vegetation composition and soil conditions vary considerably within the plot, and that was taken into account in the simulations. According to the formulation of the EFIMOD3 system of models, the computational experiments took into account the spatial heterogeneity of the distribution of tree roots, depending on species-specific features and the location, size, and age of the trees. The spatial distribution of root biomass in turn determined spatial differences in the autotrophic respiration rates calculated in EFIMOD3 and the amount of root exudates input into the EMR model. In addition, the spatial structure, species composition, and size class distribution of the simulated stand determined spatial differences in the input of species-specific fractions of above- (needles and leaves, branches, and stems) and belowground (fine and coarse roots) litter, as well as soil hydrothermal conditions, which influenced the pools and mineralization rates of SOM in EFIMOD3.

One of the aims of our study was to evaluate the contribution of EMR to soil respiration, and therefore, our test model simulations assumed a separate calculation of the total heterotrophic, autotrophic, and EMR soil respiration. In previous versions of the EFIMOD3 model system, EMR was not explicitly simulated, but these models were calibrated using the experimental data on total heterotrophic soil respiration, which implicitly included EMR respiration. We calculated autotrophic (tree roots) and heterotrophic (all soil biota) respiration using the EFIMOD3 model system described in detail in [68]. Briefly, autotrophic respiration is calculated as the sum of plant growth respiration (the fixed fraction of NPP allocated to belowground organs) and maintenance respiration (estimated with a fixed fraction of belowground biomass). Heterotrophic respiration in EFIMOD3 is calculated as the sum of carbon dioxide emissions during all stages of mineralization of soil organic matter. Ectomycorrhizal respiration was calculated using the EMR model as described above. Finally, non-mycorrhizal soil heterotrophic respiration was calculated as the difference between total soil heterotrophic respiration and EMR respiration. In addition, we compared the amount of available N from SOM decomposition calculated by EFIMOD3 and the amount of ammonium N (N-NH_4_) calculated via the EMR model.

All calculations were performed on a simulation grid of 100 × 100 m, divided into 10,000 cells of 1 × 1 m, for a period of 5 years with a daily time step. This allowed us to assess the possible variation of the indicators (components of soil respiration and N formed in FWs) both in time and in space. The temporal dynamics are reflected in the variation of values between different dates of specific years or terms of the growing season. The spatial inhomogeneity is related to the distribution of the fine roots of the trees over the simulation plot and is reflected in the ranges between the minimum and maximum values for each specific day of the simulation period.

The calculation of the amount of root exudates for the EMR model was carried out similarly to the validation, and the same C:N ratio of REs (equal to 40) was used. The temperature and moisture dynamics of the organic layer and the mineral soil were simulated using the observed data on air temperature and humidity and precipitation for 2016–2020 measured at the EMEP station “Danki” located in the reserve [87,88].

## 3. Results

The results of the sensitivity analysis (Table 3) showed that the target variable is most sensitive to the C:N ratios, especially those of EMR fungi. Other influential parameters include the respiration rates of the soil meso- and macrofauna. The analysis also showed that the model is almost linear.

**Figure 2:**
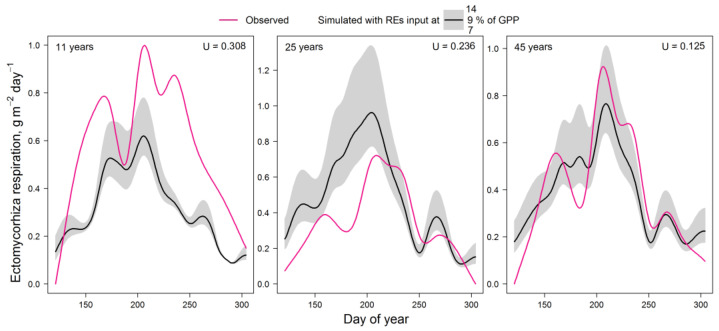
Comparison of measured and simulated seasonal dynamics of ectomycorrhizal respiration for stands of different ages. U is the Theil’s U index of inequality (for REs input at 9% of GPP).

The validation results (Figure 2) showed that the model underestimates EMM respiration in a very young plantation with low stand density. However, the model overestimates respiration in a dense 25-year-old forest. The best agreement between experimental and simulated data was obtained for the 45-year-old larch forest. The low Theil’s U index in all cases indicates the satisfactory results of this validation.

Other metrics of model agreement with experimental data for REs input at 9% of GPP are shown in Table 4.

**Figure 3:**
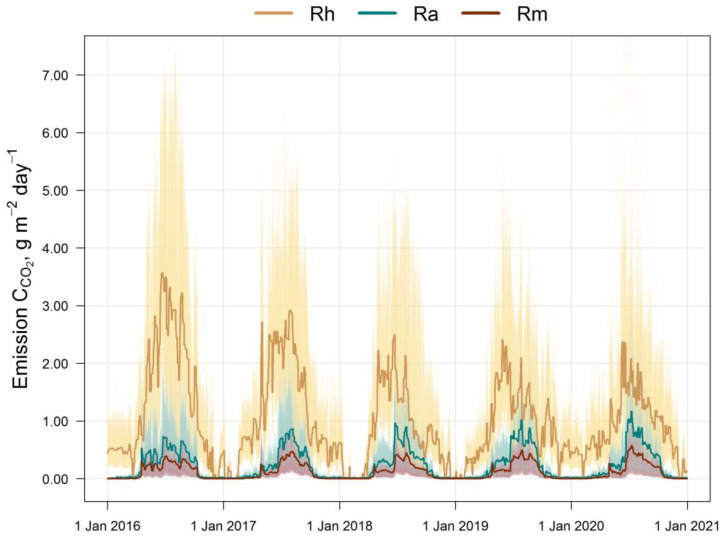
Simulated soil fluxes of C-CO_2_ for the simulation plot (10,000 cells): Rh is the heterotrophic respiration, Ra is the autotrophic respiration (tree roots), and Rm is the ectomycorrhizal respiration. The thin lines indicate the variation and bold lines indicate the mean values over the simulation plot. The data show high spatial variability (thin yellow lines) of all C-CO_2_ fluxes with maximum values in summer, as well as comparable values of Ra and Rm fluxes.

**Figure 4:**
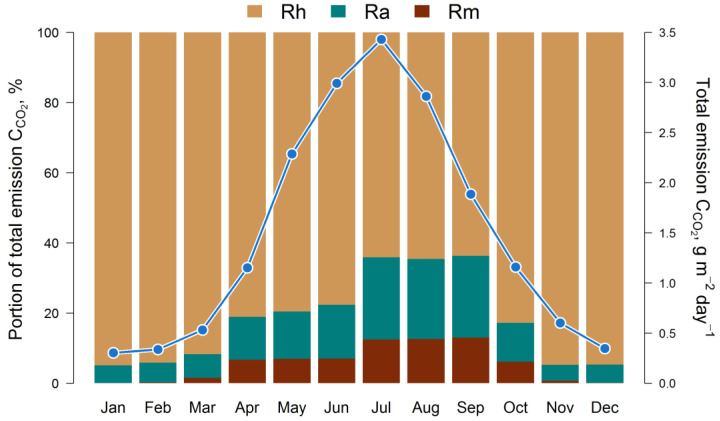
Simulated intra-annual dynamics of total soil respiration (monthly averages for 5 years) and the ratio of mycorrhizal (Rm), autotrophic (Ra), and heterotrophic (Rh) fluxes of C-CO_2_. The share of Rh in the total C-CO_2_ flux varies between 64% and 95%, with a maximum in winter. The Rm has a maximum in summer, when it is up to 13% of the total respiration and up to 56% of the Ra flux.

The results of the model testing with the estimation of the 5-year cycle of soil C-CO_2_ emission fluxes (autotrophic, mycorrhizal, and heterotrophic) are presented in Figure 3. All calculated fluxes show a remarkable temporal and spatial variation. The maximum of all fluxes corresponds to the middle of the growing season (July). The main contribution to the total soil respiration is made by heterotrophic respiration, whose share varies from 45% in summer to 95% in winter, against the background of a corresponding decrease in the average total flux of C-CO_2_ from 3.5 to 0.4 g m^−2^ day^−1^ from July to December (Figure 4).

In the first half of the growing season (April–June), ectomycorrhizal respiration is estimated via the EMR model at an average of 0.25–0.3 g m^−2^ day^−1^ (in terms of C), and at 0.35–0.4 g m^−2^ day^−1^ in the second half (July–September). The dynamics of ectomycorrhizal respiration rates correlate with the autotrophic (stand) respiration rate, which follows from the general statements of the EMR model. The 5-year average estimates for the growing season (April–October) show that EM respiration can reach 35–60% of the autotrophic C-CO_2_ flux and up to 15% of the respiration of soil bacteria and non-mycorrhizal fungi.

**Figure 5:**
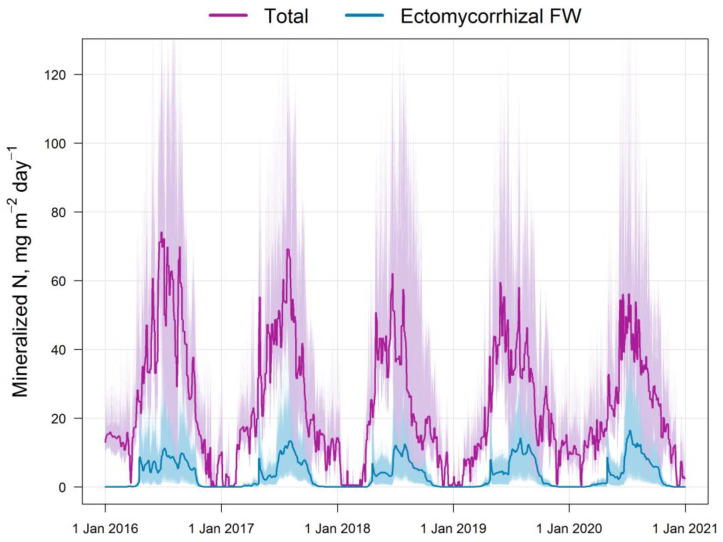
Simulated dynamics of the total flux of available N calculated through EFIMOD3 (Total) and N-NH_4_ calculated through the EMR model (Ectomycorrhizal FW) for the simulation plot (10,000 cells). The thin lines indicate the variation, whereas the bold lines indicate the mean values over the simulation plot. The data presented illustrate the seasonal dynamics and high spatial variability of N fluxes in forest soil.

**Figure 6:**
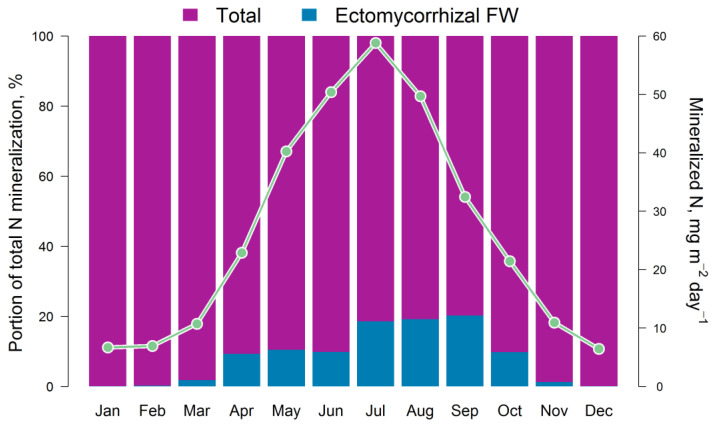
Intra-annual dynamics of the total flux of available N in the soil (monthly averages for 5 years) and the ratio of available (Total) and ammonium N fluxes (Ectomycorrhizal FW) calculated via the EFIMOD3 and EMR models, respectively. During the growing season (April–October), the ammonium N flux formed by ectomycorrhizal FW ranges from 10% to 25% of the available N formed due to the SOM mineralization.

The results of the comparison of the N available to plants calculated via the EFIMOD3 and EMR models are shown in Figure 5. The obtained patterns of dynamics of these indicators are similar to the dynamics of C-CO_2_ fluxes mentioned above. The amount of ammonium N (N-NH_4_) formed in soil food webs during the summer months (June–August) is estimated through the EMR model to be 5–10 mg m^−2^ day^−1^ on average (in terms of N) with a range of variation from close to zero values to 40 mg m^−2^ day^−1^. The 5-year average estimates for the growing season (April–October) show that the pool of ammonium N formed as by-products of micro- and mesofauna activity in soil food webs can reach 10–12% of the total pool of the available N formed during SOM mineralization (Figure 6). It should be noted that another noticeable fraction of EMR-derived N reaches trees via the decomposition of the labile SOM pool of faunal excreta. In addition, we found that the total C of the EMM-derived casts of micro- and mesofauna of the food webs varied between 0.1 and 0.3 t ha^−1^ during 3 months of summer.

## 4. Discussion

### 4.1. Theoretical Background and Structure of the Model

Currently, there are two main concepts of the symbiotic interactions between plant and EMR [27,89]: (i) mutualistic with a direct exchange of resources between plant and fungi (“carbon by nitrogen”), and (ii) the indirect effect of EMR on the host plant with a specific pathway of N return from EMM to plant roots via EMM decomposition. The proposed model is an attempt to quantify the second approach, with EMR as an obligate biotroph that consumes C and all N from REs, as well as additional N derived (“mined”) from SOM, for its own growth only, thus actually acting as a commensalist. N is returned to the plant through the fast cycle of EMM transformation and decomposition by the biota of soil food webs, including the activity of microorganisms, micro-, and mesofauna [40]. The majority of recent EMR models describe EMM growth without this N feedback to the plant, with the exception of [4], which calculates different pathways of N acquisition.

The structure of the proposed model has been developed for integration into EFIMOD3 model system. Therefore, the model is focused on N production for tree growth, as well as C turnover and sequestration in the soil. Both tasks were solved by incorporating food webs into the model structure by establishing a previously missing N-rich “fresh and free” SOM pool of faunal excreta as a solid-phase organic matter not yet bound to R_2_O_3_, Ca, and clay minerals.

### 4.2. Sensitivity Analysis, Validation, and Testing

The sensitivity analysis revealed a leading role of the C:N ratio in root exudates, EMM, and soil biota biomass, which seem to be the main drivers in the EMR symbiosis, as was theoretically justified earlier [14,15]. In principle, the same effect has already been described in the simulation model of the rhizosphere priming effect [21].

In the model validation, the underestimation of simulated EMM respiration (compared to observed values) in sparse tree plantations (aged 11 years), where soil patches without tree roots may occur, may be a result of an overestimation of respiration for the whole experimental plot. Other reasons could be both the high metabolic activity of young trees and differences between actual and simulated soil properties on the experimental plot, as the soil was assumed to be the same in all simulation scenarios. The overestimation of simulated respiration in the 25-year-old stand was apparently related to an unaccounted effect of a colder microclimate in dense stands of this age [90,91]. The overestimation of simulated respiration in early spring and late autumn may be related to the unaccounted influence of short daylight hours. The results of test simulations in a frame of the EFIMOD3 system also showed the same order of magnitude as the experimental data [92,93,94]. Moreover, they demonstrate new possibilities of the proposed model for the quantification and specification of carbon and nitrogen flows that were not previously accentuated in forest ecosystems research.

### 4.3. Role of EMR and Food Webs in the Soil Organic Matter Dynamics

EMM produces large amounts of DOC during N mining [22]. This occurs because EMM first depolymerizes large molecules into soluble, low-molecular-weight OM. EMM then extracts and uses only the N-rich OM and does not consume the rest as other saprotrophic microorganisms do. The proposed model is, to our best knowledge, the first to include this specific EMR-derived DOC pool in the calculation of SOM dynamics.

An important detail is that the prevailing concept now assumes that microbially derived DOC is a significant source of SOC sequestration on mineral surfaces [95]. However, soil microorganisms produce DOC for their own use during the initial stage of nutrient consumption, i.e., during SOM decomposition [41]. Therefore, microorganisms have a limited capacity for free DOC production and consequently for SOM formation (in a process of SOM decomposition). Logically, DOC in forest soils can be mainly represented by the mycorrhiza-derived substances obtained from the specific SOM depolymerization during N mining.

Furthermore, if the activity of the fauna of food webs is excluded from the processes of EMM transformation and decomposition (as in the vast majority of biogeochemical and microbiological SOM decomposition models), the capacity of EMR for SOM sequestration will be significantly underestimated. In this case, the intermediate product of decomposition will be the small amount of DOC that is bound only by clay minerals. Soil minerals have a limited sorption capacity with the possibility of “carbon saturation” [95] compared to the unlimited potential of excretes’ and casts’ formation by micro- and mesofauna. These are micromorphologically visible structures [96,97] that can be further protected in soil by R_2_O_3_ or Ca^2+^.

The calculated total production of faunal casts (in terms of C) can reach 0.1–0.3 t ha^−1^ for three summer months in a temperate broadleaved–coniferous forest, as was found in the model experiments. This value is only three times lower than the cast production by earthworms, which varies from 0.1 to 1.0 t ha^−1^ (in terms of C) during the growing season (adopted from [98,99]). The casts from EMM-derived litter are processed by the gut microbiomes of the soil micro- and mesofauna. Apparently, they play the same role as the “carbon trap” mediated by earthworms [100].

EMM was rather rapidly consumed by soil biota [45,101], with C being returned to SOM as the above-mentioned solid-phase pool of faunal metabolites. They are used for organic C sequestration and also to increase the amount of N available to roots in the case of this young N-rich organic matter mineralization. The balance between these two processes depends on biological and environmental factors. This cycle has not yet been considered in mycorrhizal models and is, therefore, a task for future research.

### 4.4. Role of EMR in Nitrogen Dynamics and Supply for Tree Growth

The excess carbon of root exudates forces EMM to mine N for biomass growth from protected stable SOM with a low C:N ratio. The model allows the calculation of the amount of N mined for the growth of short-living EMM. It provides an opportunity for a more accurate assessment of the contribution of EMR to the N budget of the forest ecosystems. The formation of available ammonia N in food webs is an additional factor in the positive effect of EMR with the significant increase in tree growth. This occurs because some excess N is produced during the consumption of microorganisms (including EMM) by soil fauna, as a significant proportion of food C is used for faunal respiration [48,49,51]. The dominance of “mined” organic N and ammonia in EMR and soil are specific features of EMR nitrogen budget as previously characterized [25,89].

### 4.5. Aspects of the EMR Functioning

From an ecological point of view, the results of this model validation and testing prove that the representation of mycorrhiza as a “commensalist” can be a main pattern to describe the functioning of EMR in forest ecosystems. EMR “works for itself”; however, its positive effect on tree growth can be explained by the rapid transformation and decomposition of EMM by all soil biota, including microorganisms and fauna of soil food webs. In other words, the mycorrhizal symbiosis can be represented as a triad of biological actors: “roots–mycorrhiza–all soil biota” [40]. They are structurally, functionally, and perhaps evolutionarily linked in the forest ecosystem. We understand that this model approach reflects only some aspects of EMR life and functioning that are more important for EMR contribution to the biological cycle and tree growth in forest ecosystems. New data are needed to specify the processes of N mining, DOC formation, food web activity, and natural mortality of EMM.

In the described model, there are no pathways for a direct resource exchange between the host plant and ectomycorrhiza (C from plant for N from ectomycorrhiza). Nevertheless, the satisfactory results of the model validation show that the positive effect of EMR on the plant, with the production of additional N and SOM sequestration via faunal casts, can only be achieved during the fast cycle of transformation and decomposition of EMM litter by all soil biota.

## 5. Conclusions

The proposed model of ectomycorrhiza in forest ecosystems is designed to be incorporated into EFIMOD3 and possibly other forest models. Therefore, the role of EMR in the N budget and SOM dynamics in forest ecosystems is emphasized. The model is based on the concept of an indirect positive effect of EMR on plant growth due to the rapid decomposition of short-living EMM, which represents a dominant belowground litter pool in forest soils. The model allows for the assessment of EMM growth, N mining and release for plant nutrition, and SOM formation. The growth of EMM is determined by the amount and the C:N ratio of root exudates. The mycorrhizal N mining of SOM is calculated in the model only for EMM’s own use, with a significant amount of dissolved organic matter as a by-product. The decomposition of EMM by all soil biota of the food webs allowed the estimation of (i) the available N-NH_4_ that can be directly used by plants and (ii) the solid-phase by-product of faunal metabolism, which is a pool of fresh N-rich organic matter that has never been represented in EMR models. The validation and testing of this model demonstrated the appropriateness of the theoretical background used in this model.

## Figures and Tables

**Table 1:** Input and output parameters of the ectomycorrhiza model.

Parameters	Dimension
Input parameters	
Total soil C	kg m^−2^
Total soil N	kg m^−2^
Root exudates input in terms of C	kg m^−2^ day^−1^
Root exudates C:N ratio	dimensionless
Extramatrical mycelium biomass in terms of C	kg m^−2^
Ectomycorrhiza C:N ratio	dimensionless
Microfauna biomass in terms of C	kg m^−2^
Microfauna C:N ratio	dimensionless
Output parameters	
Total C-CO_2_ emission at ectomycorrhiza functioning	kg m^−2^ day^−1^
N produced at SOC mining via ectomycorrhiza	kg m^−2^ day^−1^
DOC produced at SOC mining via ectomycorrhiza	kg m^−2^ day^−1^
Sum of microfauna excreta and all necromass C for labile SOC pool	kg m^−2^
Sum of N produced for labile SOC pool	kg m^−2^
N-NH_4_ excreted by food webs fauna	kg m^−2^

**Table 2:** Variables of the ectomycorrhiza model.

Parameters	Valid Range	Default Value
REs assimilation rate via EMM	0.00–1.00	1.00
Coefficient of competition for REs between EMR (fungi) and rhizosphere priming (bacteria)	0.00–1.00	1.00
EMM C:N ratio	9.00–25.00	13.10
EMM respiration coefficient	0.50–0.90	0.70
EMM production/growth coefficient	0.10–0.50	0.30
EMM consumption coefficient	0.00–0.25	0.24
EMM mortality coefficient	0.00–0.25	0.01
Micro- and mesofauna C:N ratio	8.00–11.00	10.00
Macrofauna C:N ratio	6.00–10.00	8.00
Micro-, meso-, and macrofauna respiration coefficient	0.50–0.80	0.60
Soil fauna * consumption coefficient	0.00–0.25	0.20
Soil fauna * excretion coefficient	0.10–0.40	0.20
Soil fauna * mortality coefficient	0.00–0.25	0.01

Note: Data from [5,21,48,52,53,70,71,72] were used. Production efficiency (food web terminology) corresponds to carbon use efficiency (CUE, microbiological terminology). All variables are dimensionless. * The same coefficients are used for soil micro-, meso-, and macrofauna.

**Table 3:** Analysis of model sensitivity to uncertainty in input parameters.

Parameter	Parameter Name	Standardized Coefficient of Linear Regression
C:N ratio of root exudates	${CN}_{RE}$	−0.245 ***
C:N ratio of ectomycorrhiza	${CN}_{EMM}$	0.723 ***
C:N ratio of soil mesofauna	${CN}_{MEF}$	−0.344 ***
C:N ratio of soil macrofauna	${CN}_{MAF}$	−0.164 ***
Respiration rate of ectomycorrhiza	$k_{EMM}^{R}$	0.238 ***
Mortality rate of ectomycorrhiza	$k_{EMM}^{mort}$	−0.0553 ***
Consumption rate of ectomycorrhiza	$k_{EMM}^{cons}$	0.0730 ***
Mortality rate of soil micro- and mesofauna	$k_{MEF}^{mort}$	−0.0126 ***
Consumption rate of soil micro- and mesofauna	$k_{MEF}^{cons}$	0.0167 ***
Respiration rate of soil micro- and mesofauna	$k_{MEF}^{R}$	0.326 ***
Respiration rate of soil macrofauna	$k_{MAF}^{R}$	0.142 ***
Intercept		~0
R^2^		0.921

Note: *** indicates the significance level (*p* < 0.001).

**Table 4:** Metrics for model agreement with experimental data.

Metric	Formula	11 Years	25 Years	45 Years
Mean Squared Error	$\frac{1}{n}\sum_{i=0}^{n}{\left(x_{i}-y_{i}\right)}^{2}$	0.0936	0.0508	0.0125
Root Mean Squared Error	$\sqrt{\frac{1}{n}\sum_{i=0}^{n}{\left(x_{i}-y_{i}\right)}^{2}}$	0.306	0.225	0.112
Mean Absolute Error	$\frac{1}{n}\sum_{i=0}^{n}\left|x_{i}-y_{i}\right|$	0.269	0.179	0.0936
Symmetric Mean Absolute Percent Error	$\frac{2}{n}\sum_{i=0}^{n}\frac{\left|x_{i}-y_{i}\right|}{{|x}_{i}|+|y_{i}|}$	0.628	0.464	0.284
Mean Squared Log Error	$\frac{1}{n}\sum_{i=0}^{n}{\left(\ln\left(1+x_{i}\right)-\ln\left(1+y_{i}\right)\right)}^{2}$	0.0410	0.0224	0.00615
Root Mean Squared Log Error	$\sqrt{\frac{1}{n}\sum_{i=0}^{n}{\left(\ln\left(1+x_{i}\right)-\ln\left(1+y_{i}\right)\right)}^{2}}$	0.202	0.149	0.0784
Relative Squared Error	$\frac{\frac{1}{n}\sum_{i=0}^{n}{\left(x_{i}-y_{i}\right)}^{2}}{\frac{1}{n}\sum_{i=0}^{n}{\left(x_{i}-\bar{x}\right)}^{2}}$	1.589	1.355	0.228
Root Relative Squared Error	$\sqrt{\frac{\frac{1}{n}\sum_{i=0}^{n}{\left(x_{i}-y_{i}\right)}^{2}}{\frac{1}{n}\sum_{i=0}^{n}{\left(x_{i}-\bar{x}\right)}^{2}}}$	1.260	1.164	0.477
Relative Absolute Error	$\frac{\frac{1}{n}\sum_{i=0}^{n}\left|x_{i}-y_{i}\right|}{\frac{1}{n}\sum_{i=0}^{n}\left|x_{i}-\bar{x}\right|}$	1.312	1.139	0.471

## Data Availability

The data are available from the authors upon reasonable request.
